# Supplementary material for: Hemocyte-targeted gene expression in the female malaria mosquito using the hemolectin promoter from Drosophila
Source: Insect Biochem Mol Biol. 2020 May;120:103339. doi: 10.1016/j.ibmb.2020.103339 (PMC7181189; doi:10.1016/j.ibmb.2020.103339)
Supplement: Multimedia component 1 [file mmc1.pdf]

Supplementary Material

Hemolectin orthology assessments across arthropods

Identification of putative orthologs of the *Drosophila melanogaster hemolectin* gene in arthropod species with sequenced and annotated genomes proceeded by searching genomic resources hosted at VectorBase release VB-2019-08 (Giraldo-Calderón et al.) and the OrthoDB hierarchical catalog of orthologs versions 9 and 10 (Zdobnov et al.). First, VectorBase was searched using the FlyBase gene identifier for *Hemolectin* (FBgn0029167), to identify the GeneTree (family of homologous genes) of which it is a member, GeneTree VBGT00190000010751. GeneTrees are built automatically by VectorBase using the EnsemblCompara pipeline (Vilella et al.) that uses the longest protein-coding translation of each gene with searches of the TreeFam (Schreiber et al.) library of sequence profiles followed by multiple sequence alignments with combinations of various aligners and then tree building with TreeBeST (Vilella et al.). This revealed orthologs in other Brachycera flies (*Musca domestica* and five *Glossina* species), three non-holometabolous insects (*Rhodnius prolixus*, *Cimex lectularius*, and *Pediculus humanus*), and the tick, *Ixodes scapularis* (Figure S3), as well as potential orthologs in two sandflies (incomplete gene models make orthology difficult to confirm with confidence). Second, the *D. melanogaster* Hemolectin protein sequence was searched using tBLASTn against all nucleotide data (genomes, transcriptomes, ESTs, etc.) from all species hosted by VectorBase. These searches confirmed the results from the automated GeneTree ortholog identification, i.e. several matches to mosquito genes that encode von Willebrand factor domains but no *Hemolectin* orthologs in mosquitoes. For a broader perspective across arthropods, searches of OrthoDB identified *Hemolectin* orthologs in many different groups including additional non-mosquito Diptera, as well as in Lepidoptera, Coleoptera, Hymenoptera, Hemiptera, Thysanoptera, Isoptera, Blattodea,

Ephemeroptera, Odonata, Diplura, Collembola, Crustacea, Myriapoda, and Arachnida, but no orthologs in any mosquito species. These analyses strongly support the evolutionarily-rare loss of the *Hemolectin* gene from the genome of the last common ancestor of mosquitoes.

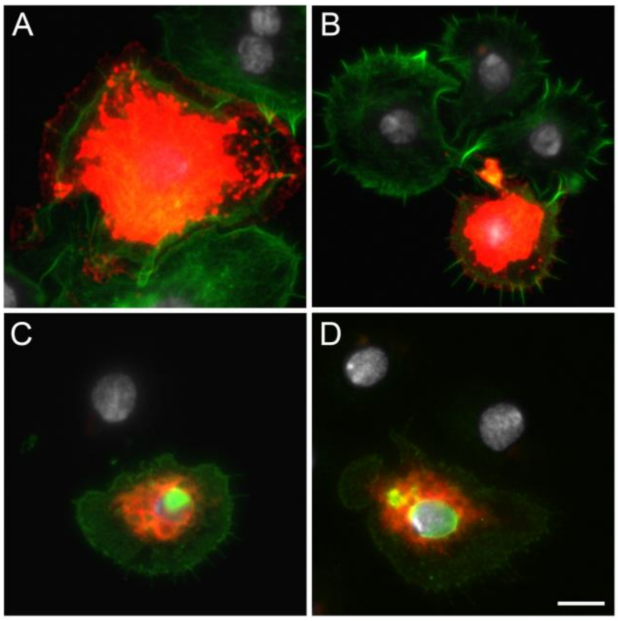

Figure S1. Hemocytes collected from *hml*>mCD8::Cherry blood fed females. (A, B) Over exposition of pictures I and J (Figure 2) showing Cherry at the cell membrane. Red: endogenous mCD8::Cherry; Green: Phalloidin; White: DAPI staining. (C, D) Anti-mCD8 antibody staining. Red: endogenous mCD8::Cherry; Green: anti-mCD8; White: DAPI staining. Scale bar is 5 µm.

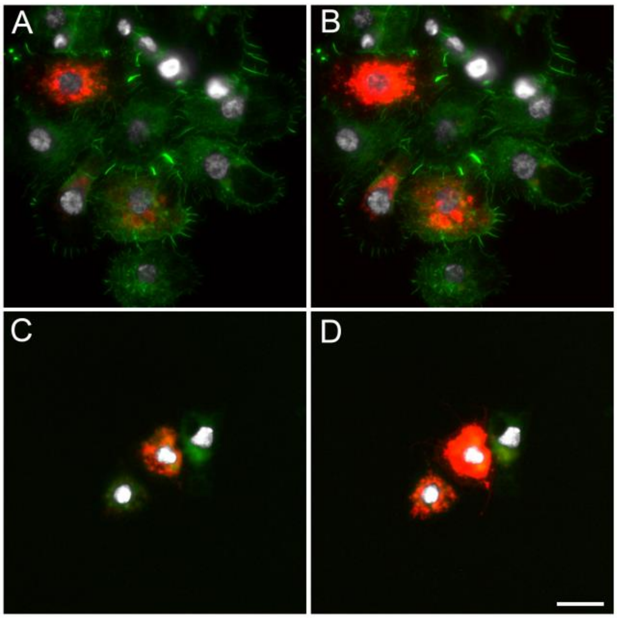

Figure S2. Hemocytes collected from *hml*>mCD8::Cherry blood fed females. B and D are highly exposed pictures from A and B respectively. Red: endogenous mCD8::Cherry; Green: Phalloidin; White: DAPI staining. Scale bar is 10 µm.

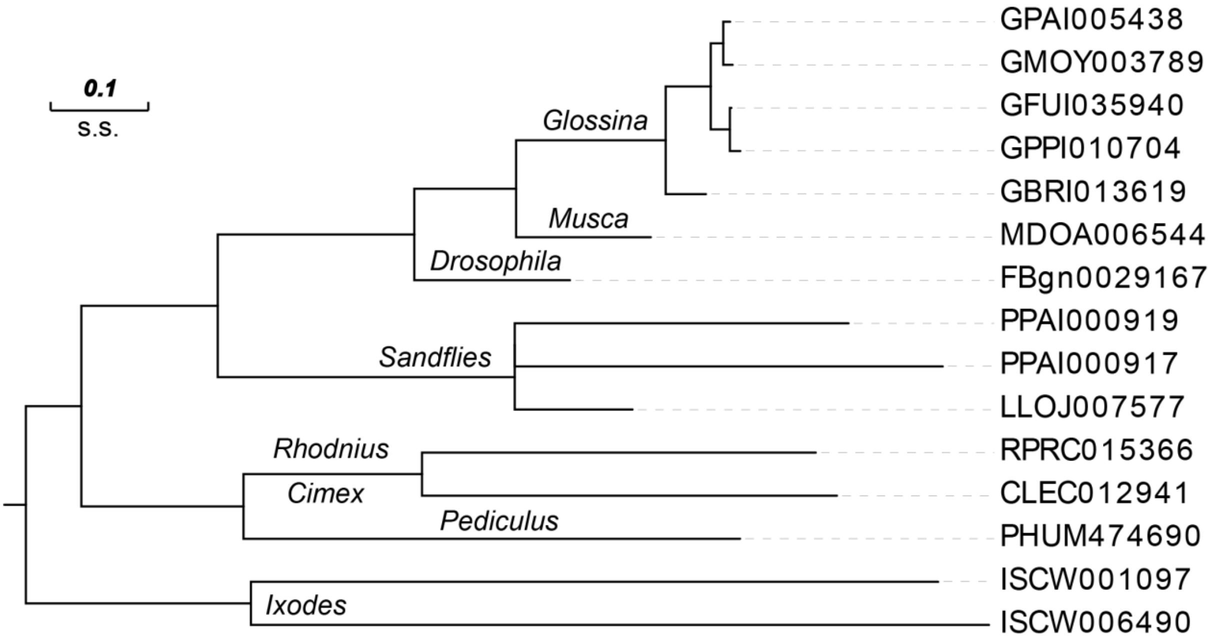

Figure S3. *Hemolectin* gene tree from VectorBase. Orthologs of *Drosophila melanogaster hemolectin* were found in other Brachycera flies and outgroup non-holometabolous insects and the *Ixodes* tick. Note that the two *Phlebotomus papatasi* genes are likely fragments of a single gene, similarly for the two *Ixodes scapularis* genes. No orthologs were found in any of the mosquito species at VectorBase.

Table S1. Sequence of primers used in the study.

**Gene**	**Forward primer**	**Reverse primer**
S7	AGGCGATCATCATCTACGTGC	GTAGCTGCTGCAAACTTCGG
gal4	TGAAGCTACTGTCTTCTATCG	GTAGCGACACTCCCAGTTG
cherry	tagcagccagatctGTCGACGGTATCGATAAGC	tagcagccTCTAGATTACTTGTACAGCTCGTCCATGC
